# DNA barcoding in quarantine inspection: a case study on quarantine insect monitoring for Lepidoptera obtained through quarantine inspection on foreign vessels

**DOI:** 10.1080/23802359.2018.1536447

**Published:** 2018-12-27

**Authors:** Tae Hwa Kang, Sunam Kim, Ki-Jeong Hong, Heung Sik Lee

**Affiliations:** a Bio Control Research Center, Jeonnam Bioindustry Foundation, Gokseong-gun, Korea;; b Department of Plant Medicine, Sunchon National University, Suncheon-si, Korea;; c Plant Quarantine Technology Center, Animal and Plant Quarantine Agency, Gimcheon-si, Korea

**Keywords:** DNA barcode, Lepidoptera, quarantine pest, vessel, quarantine inspection

## Abstract

We conducted quarantine insect species diversity monitoring using DNA barcoding with 517 lepidopteran samples that were obtained from quarantine inspections of foreign vessels entering Korea. For species delimitation and species identification of the analyzed samples, we applied a 2% cutoff rule. Consequently, 145 (368 samples) were considered taxonomically identified. Therefore the number of samples that were identified to the species level was relatively low, at approximately 71%. Thirty of 145 species were not known in Korea, three, i.e., *Noctua pronuba* (Noctuidae), *Orthosia hibisci* (Noctuidae), and *Pieris brassicae* (Pieridae), were checked as ‘Regulated pests’ in Korea.

## Introduction

Biosecurity is one of the most important issues facing the international community and encompasses the protection against any risk of biological harm, such as the threatening of ecosystem stability, producer livelihoods, and consumer confidence (Cock 2003; Armstrong and Ball 2005). Specifically, invasive species are among the important factors that facilitate risk and are strongly shaped by trends in human trade and transport (Armstrong and Ball 2005; Hulme 2009). Biological invasions can be a major cause of declines and extinctions of native species (Clavero and García-Berthou 2005; Ricciardi et al. 2011). Considering only economic losses, the costs associated with environmental damage and loss in productivity due to the inflow invasive species have been estimated to total $120 to $137 billion per year in the United States (Pimentel et al. 2005). The International Plant Protection Convention has already recognized the necessity of international cooperation to control pests of plants and plant products and to prevent their international spread and introduction into endangered areas (IPPC 1997). However, one of the main recognized weaknesses of prevention plans is the difficulty of predicting new invasive alien species, which limits the implementation of appropriate risk management strategies (Parliamentary Commissioner for the Environment 2000; Armstrong and Ball 2005). A critical aspect of prediction and monitoring is the ability to accurately identify any intercepted specimen to the species level (Armstrong and Ball 2005). The management of risks posed by potential invasive species requires, at a minimum, the ability to recognize those species (Darling and Blum 2007).

The Animal and Plant Quarantine Agency of Korea treats and manages 1552 plant pest species as regulated pests (60 quarantine pest species and 1492 regulated non-quarantine pest species) (Animal and Plant Quarantine Agency, http://www.qia.go.kr). Moreover, the species identification manuals for plant pests are based on morphological data provided in the plant pest information database of the intranet of that institute (Pest Information System: PIS). However, morphological methods appear to suffer several problems in terms of the management of invasive species, which requires rapid and accurate species identification but with little access to expert taxonomic skills. Also, the difficulty of identifying species in their early life stages based on morphological criteria is well known (Besansky et al. 2003; Darling and Blum 2007). Long-term research strategies are required to address the deficiencies in the existing taxonomic keys to address morphologically indistinct immature life stages, cryptic species and damaged specimens (Armstrong and Ball 2005). To solve these problems, molecular methods have been used for species identification (Darling and Blum 2007). Among these methods, the application of DNA barcoding is increasing in popularity for the species identification (Armstrong and Ball 2005; Darling and Blum 2007).

DNA barcoding is an identification method based on comparing the 5-prime region (658 base pairs) of the cytochrome c oxidase I (COI) gene with those in established sequence databases such as the NCBI GenBank or BOLD system (Hebert et al. 2003; Ratnasingham and Hebert 2007). Various animal taxa have been analyzed around the world, and a total of 164,701 species are represented in BOLD (http://www.boldsystems.org, accession date: 26. October 2015). Among the insect taxa, Lepidoptera is relatively well characterized (Janzen et al. 2005; Hajibabaei et al. 2006; Lukhtanov et al. 2009; Hebert et al. 2010; Hausmann et al. 2011; Dincă et al. 2011). Regarding quarantine pests, Armstrong and Ball (2005) performed DNA barcoding analysis of both the lymantrid tussock moth and tephritid fruit fly families and enabled the utilization of DNA barcodes in quarantine inspection. Although this also highlighted that there can be limitations for distinguishing closely related species. Subsequently, various other invasive species have been analyzed with DNA barcoding (Scheffer et al. 2006; deWaard et al. 2010; Briski et al. 2011; Bennett et al. 2011; Crawford et al. 2011; Nagoshi et al. 2011; Dejean et al. 2012; Smith et al. 2012; Meeyen et al. 2014; Kang et al. 2015).

The goal of the present study was to identify the lepidopteran insects obtained through quarantine inspection on the quarantine insects using DNA barcoding. Based on the results, we attempted to assess the species diversity of the lepidopteran insects that arrive into Korea via shipping vessels towards the construction of a DNA barcode database for the PIS of the Animal and Plant Quarantine Agency of Korea towards the future species identification of quarantine pests in the future. Finally, based on the regulated pests identified in the present study, we suggest closer monitoring of the introduction routes.

## Materials and methods

### Sample collection

A total of 581 samples of quarantine insects were obtained through random searching on container contents of foreign shipping vessels that came into Korea via the International Plant-quarantine Accreditation Board between March and November in 2014 (Supplementary Table S1). All samples were deposited in the collection room of Plant Quarantine Technology Center, Animal and Plant Quarantine Agency, Korea. The specimens were dried for morphological analysis, and two or three legs were subsequently removed for genomic DNA extraction. Collection data labels were created listing the inspection seaport, name of the vessel, navigation route, identity no. of the vessel, live/dead, and inspection date (Supplementary Table S1).

### Genomic DNA extraction, polymerase chain reaction, and sequencing

The genomic DNA of each sample was extracted from the legs using a Dneasy^®^ Blood & Tissue Kit (Qiagen, Leipzig, Germany) according to the user manual. For the polymerase chain reaction (PCR) amplification of the COI barcode region, we created a mixture of a LCO1490 + HCO2198 primer set (Folmer et al. 1994) and AccuPower^®^ Pfu PCR Premix (Bioneer, Daejeon, Korea) with the following composition: 1 μl template DNA, 1 μl forward primer (LCO1490), 1 μl reverse primer (HCO2198), 17 μl distilled water, and Pfu PCR Premix. Amplification performed with a GenMax Tc-s-B (BIOER, Hangzhou, China) with the following conditions: step 1: 94 °C, 5 min, 1 cycle; step 2: 94 °C, 20 sec; 53 °C, 20 sec; and 72 °C, 30 sec for 35 cycles; and step 3: 72 °C, 5 min for 1 cycle. The amplification of single PCR products was verified with electrophoresis using 1% agarose gel. Subsequently, the PCR products were cleaned up with purification kit, ExoSap-IT^®^ PCR Product Cleanup (Affymetrix USB, Santa Clara, CA, USA) and sequenced in both directions using above primers with 3730xl DNA analyzer (Thermo Fisher Scientific, Waltham, MA, USA) by the DNA sequencing service company Macrogen (Seoul, Korea). The obtained sequences were submitted to NCBI GenBank after eliminating the primer sequences. The DNA barcodes of the 517 lepidopteran quarantine insects were submitted to the NCBI GenBank, and we were provided with the accession numbers KT988343 to KT988859 (Supplementary Table S2) and the photos of the voucher specimens for DNA barcoding (Supplementary Figure S1).

### DNA barcoding, species delimitation, and species identification

The obtained DNA sequences were aligned with Clustal W in MEGA6 (Tamura et al. 2013), and then the sequence divergence and neighbor-joining tree were estimated in MEGA6 (Tamura et al. 2013). The DNA barcode of each sample was treated as a molecular operational taxonomic unit (MOTU). The sequence divergence among the MOTUs was estimated based on the Kimura-2 parameter (K-2-P). For species delimitation of the MOTUs, we applied a 2% cutoff rule (Hebert et al. 2003; Hebert et al. 2004). BLAST search of NCBI Genbank and Identification Request of BOLD System version 3.0 were used for taxonomic identification. The MOTUs showing below 98% similarity were identified as family level. Finally, we conducted morphological re-checking on the molecular identification results at para-taxonomic level (Supplementary Table S2).

## Results and discussion

### DNA barcoding of lepidopterans obtained by quarantine inspection

DNA barcodes were obtained from 534 of 581 quarantine insect samples as follows: 3 Hemiptera individuals, 1 Neuroptera, 8 Coleoptera, 1 Diptera, 4 Hymenoptera, and 517 Lepidoptera. As the greatest number of barcodes was available for the order Lepidoptera these were used for this study of quarantine insect diversity monitoring. The sequence divergence of the lepidopteran specimens ranged from 0% to 22.5%. The frequency of the K-2-P divergence showed the highest value (4505 comparisons) at 13.3% (Supplementary Figure S2).

### Species delimitation and identification

**Figure 1.**
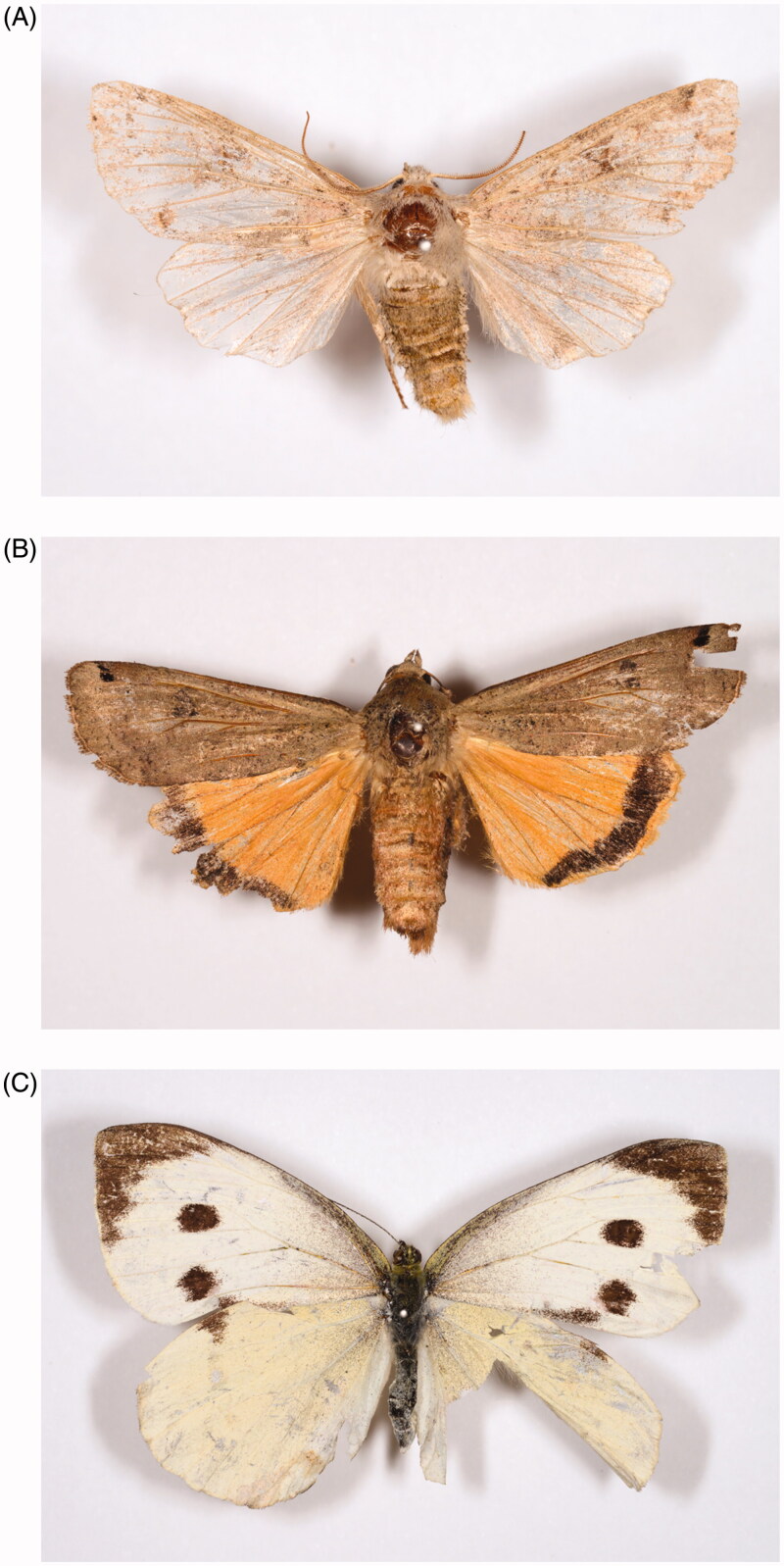
Three regulated quarantine pests detected in this study. (A) *Orthosia hibisci*; (B) *Noctua pronuba*; (C) *Pieris brassica*.

Among the 517 quarantine lepidopteran insect samples, 368 were taxonomic identified at species level, 149 were at family level (Supplementary Table S2). Subsequently, the samples were identified via 'BLAST search of NCBI GenBank' and 'Identification Request of BOLD system', and then classified as belonging to 20 families. Among the 214 species, 145 were identified to a taxonomic species (Table 1; Supplementary Table S2). Five hundred and seventeen analyzed samples comprised 161 Noctuidae samples, 155 Erebidae samples, 57 Geometridae samples, 42 Notodontidae samples, and fewer than 30 samples of each of 16 families (Table 1). Based on these results, the Noctuidae and Erebidae species exhibited a higher probability of being introduced from their origin regions into Korea relative to the other families. Total of 145 putative species were identified with BLAST searches of NCBI GenBank and Identification Request of BOLD System. One hundred and fifteen species among the 145 species identified at species level were found in the Korean lepidopteran fauna, but 30 species were not known in Korea (ESK and KSAE 1994; Paek et al. 2010; Table 2). Among 30 species not known in Korea, number of species of Erebidae and Noctuidae were more than other families as 11 species and seven species, respectively. Among the 30 species that were not known in Korea, we detected three regulated species, *Noctua pronuba*, *Orthosia hibisci*, and *Pieris brassica* (Table 2; Figure 1). Two species, *Noctua pronuba* and *Pieris brassica* were dead, but the *Orthosia hibisci* sample was alive. Therefore, we suggest that the introduction routes of the three regulated species should be monitored and this is discussed further below.

**Table 1.** Number of putative species of the 517 lepidopteran insects obtained through quarantine inspection by DNA barcoding.

Family	No. of individual	No. of species	No. of identification at species level (no. of identified individual)
Arctiidae	2	2	2 (2)
Brahmaeidae	1	1	1 (1)
Cossidae	1	1	0 (0)
Crambidae	21	11	8 (13)
Drepanidae	4	3	2 (3)
Erebidae	155	42	36 (122)
Geometridae	57	31	15 (29)
Hesperiidae	1	1	1 (1)
Limacodidae	9	4	3 (8)
Noctuidae	161	64	37 (106)
Nolidae	6	5	3 (4)
Notodontidae	42	17	9 (27)
Nymphalidae	7	5	5 (7)
Pieridae	2	2	2 (2)
Pyralidae	18	6	3 (14)
Sesiidae	1	1	1 (1)
Sphingidae	23	14	14 (23)
Tortricidae	1	1	0 (0)
Uraniidae	2	1	1 (2)
Zygaenidae	3	2	2 (3)
Total	517	214	145 (368)

**Table 2.** List of the species that are not known in Korea. A total of 30 species were found through DNA barcoding.

No	Family	Identification Result	Sample No	Remarks
1	Crambidae	*Palpita quadristigmalis*	1262, 1263, 1463, 1596	
2	Erebidae	*Achaea serva*	1548	
3	Erebidae	*Asota caricae*	1443, 1530	
4	Erebidae	*Asota heliconia*	1507, 1531, 1617	
5	Erebidae	*Catocala doerrisi*	1660	
6	Erebidae	*Daddala lucila*	1362, 1645, 1670	
7	Erebidae	*Dichromia quinqualis*	1594	
8	Erebidae	*Eilema depressum*	1289	
9	Erebidae	*Eudocima phalonia*	1535, 1556	
10	Erebidae	*Hydrillodes metisalis*	1604	
11	Erebidae	*Laelia umbrina*	1233	
12	Erebidae	*Pericyma crueqeri*	1592	
13	Geometridae	*Abraxas tenuisuffusa*	1242	
14	Geometridae	*Asthena undulata*	1597	
15	Geometridae	*Odontopera aurata*	1180, 1320, 1321	
16	Noctuidae	*Athetis thoracica*	1406	
17	Noctuidae	*Leucania venalba*	1128	
18	Noctuidae	*Noctua comes*	1495	
19	Noctuidae	*Noctua pronuba*	1459	RP
20	Noctuidae	*Orthosia hibisci*	1140	RP
21	Noctuidae	*Plusia nichollae*	1499	
22	Noctuidae	*Stenhypena albopunctata*	1292	
23	Notodontidae	*Cerura erminea*	1238, 1510	
24	Nymphalidae	*Libythea lepita*	1348, 1665	
25	Pieridae	*Pieris brassicae*	1533	RP
26	Pyralidae	Arippara disticha	1210, 1293, 1399, 1431, 1432, 1433, 1438,1460, 1472, 1595, 1681	
27	Sphingidae	*Aellopos titan*	1537	
28	Sphingidae	*Psilogramma japonica*	1576, 1578	
29	Uraniidae	*Lyssa zampa*	1446, 1447	
30	Zygaenidae	*Histia flabellicornis*	1508	

RP: Regulated pests.

### Monitoring of quarantine inspection using DNA barcoding

The limitations of morphological approaches in species identification have led many researchers and managers to propose and pursue the development of DNA-based tools (such as PCR/RFLP, SSP, SSCP and DGGE, DNA barcoding, qPCR, shotgun barcoding, T-RFLP, and POA) for the monitoring of invasive species (Darling and Blum 2007). In this study, we monitored quarantine insects that were found upon the inspection of foreign vessels with DNA barcoding. The samples obtained during quarantine inspections are often seriously damaged due to being on the ship for a long time (Kang et al. 2015). Therefore, we considered that DNA barcoding might be more accurate and faster than morphological approaches. Moreover, we selected the lepidopteran insects for monitoring quarantine insects because more DNA barcodes are available for this taxa than for other insect taxa (Janzen et al. 2005; Hajibabaei et al. 2006; Lukhtanov et al. 2009; Hebert et al. 2010; Hausmann et al. 2011; Dincă et al. 2011). Nevertheless, our results revealed that the ratio of the species identified at the species level was relatively low, at approximately 71% of a total of 517 samples (368 samples at the species level and 149 samples at the family level). Although these results were acquired via the use of DNA barcode databases, notorious invaders are well represented in existing databases (Briski et al. 2011), and although genetic databases remain incomplete, DNA barcoding can be used to resolve nearly twice the number of species that can be identified by traditional taxonomy (Briski et al. 2011). Thus, we can construct an appropriate DNA barcode library for quarantine inspection using DNA barcodes obtained through this study the if 149 samples identified at the family level are identified at the species level. Moreover, accurate species identification is a principal component of invasive species management (Briski et al. 2011) and is essential for preventing the introduction of and response to any invasions (Bax et al. 2001; Briski et al. 2011). Consequently, the use of DNA barcode databases for quarantine inspection can lead to more accurate and faster species identification.

The results of this study revealed three quarantine pests: *Noctua pronuba*, *Orthosia hibisci*, *Pieris brassica*. Among these pests, two species, *Noctua pronuba* and *Pieris brassica,* were found in the vessel left from Tacoma, WA, USA, and one, *Orthosia hibisci* was detected from a vessel that passed through the Korea Yeongheung seaport. In the case of *Orthosia hibisci*, we considered that the navigation route of the vessel containing the species should be examined with the distribution of the species because the species was not known in Korea. For invasion pathway management, we must understand invasions as spatial processes and integrate different variables to generate risk maps that highlight the hotspots of invasion likelihood (Buckley 2008; Hulme 2009). Therefore, the vessels that navigated the above route must be carefully inspected in terms of the three quarantine pests.

The purpose of quarantine inspection is to prevent the spread and introduction of pests of plants and plant products (IPPC 1997). Accordingly, quarantine inspection is performed mainly in airports and seaports that are frequently visited by foreign airplanes and vessels in Korea. Nevertheless, a total of 42 exotic invasive insect species are known to be present in Korea. Among these, 10 were introduced after 2000 (Supplementary Table S3). It has been reported that it is difficult to manage these species because they had already dispersed in local areas or the entire country when the developments of the invasive species were detected by the Animal and Plant Quarantine Agency of Korea (Hong et al. 2010). These invasive pests might be introduced due to vulnerable quarantine inspection procedures in Korea, and the management programs for these species might not be performed in the early stages of introduction. Considering the previous cases (Supplementary Table S3), the prediction regarding the likelihood of invasive new alien species may lead to the implementation of appropriate risk management strategies (Armstrong and Ball 2005). To predict invasive likelihoods, quarantine pests and native species must be constantly monitored around seaports and airports. Therefore, the introduction rates of alien species might be decreased if the quarantine insects and their introduction pathways are monitored constantly.
